# Impact of diurnal temperature range on cardiovascular disease hospital admissions among Chinese farmers in Dingxi (the Northwest China)

**DOI:** 10.1186/s12872-021-02065-8

**Published:** 2021-05-22

**Authors:** Guangyu Zhai, Jintao Qi, Guorong Chai

**Affiliations:** 1 School of Economics and Management, Lanzhou University of Technology, Lanzhou, 730050 People’s Republic of China; 2 School of Management, Lanzhou University, Lanzhou, 730000 People’s Republic of China

**Keywords:** Diurnal temperature range, Cardiovascular diseases, Distributed lag nonlinear model, Poverty area

## Abstract

**Background:**

Diurnal temperature range (DTR) has been widely applied in exploring its effect on cardiovascular disease (CVD). However, few studies have investigated the correlations between DTR and CVD in poor rural areas in China. This study aimed to examine the association between DTR and CVD in rural China.

**Methods:**

A distributed lag nonlinear model was used to evaluate the relationship between DTR and CVD risk among farmers living in the city of Dingxi (Northwest China) in the period from January 1, 2016 to December 31, 2019.

**Results:**

We observed nonlinear M-patterns between the relative risk (RR) of DTR (reference: median DTR, 12 °C) and CVD hospitalizations in all subgroups. The peak RR of CVD was noticed at DTR of 6 °C (total: 1.418; men: 1.546; women: 1.403; young: 1.778; old: 2.549) and 17 °C (total: 1.781; men: 1.937; women: 1.712; young: 2.233; old: 1.798). The adverse effect of DTR on CVD risk was more pronounced in females (RR 1.438) and elderly (RR 2.034) than males (RR 1.141) and younger adults (RR 1.852) at the extremely low (5th, 4 °C) DTR. The reverse was observed at the extremely high DTR (95th, 19 °C) (male: 1.267; females: 0.993; young: 1.586; old: 1.212).

**Conclusions:**

DTR is associated with CVD morbidity. This association was more pronounced in women and elderly, but men and younger peoples at extremely high DTR (19 °C). Future measures should take DTR into account to prevent CVD among susceptible populations.

**Supplementary Information:**

The online version contains supplementary material available at 10.1186/s12872-021-02065-8.

## Background

With the increasing concern about global climate change, temperature plays a significant role in human health. It is well-established that temperature variation during certain periods is one of the most important climatic factors related to human diseases [1–4. This includes cardiovascular diseases, the leading cause of death globally [5–9]. As the worldwide average temperature rises due to the increase in minimum temperature, diurnal temperature range (DTR) has been considered an important index of climate change. DTR, defined as the intraday difference between maximal and minimal temperature, can better represent the temperature variability within one day compared with the mean temperature [10]. Prior studies have demonstrated a linear relationship between DTR and CVD mortality [11, 12]. Furthermore, the nonlinear (U-, J-, or inverted U- shaped) associations between DTR and CVD mortality have been reported in East Asia main cities, including Korea, Japan and China [13–15]. These reports and others observed an effect modification by age and gender on the association between DTR on CVD mortality, with stronger associations in females and the elderly than males and younger individuals [11, 12, 15]. Also, farmers and individuals with low-education were more susceptible to DTR than their counterparts [16, 17]. Most of these studies focused on the association between DTR and the broad categories of CVD morbidity and mortality in the urban areas. Farmers, who are more vulnerable to temperature change and have lower education levels than urban residents, were not represented enough in these studies [16]. More severe diseases related to extreme temperature have been reported in the impoverished region due to lack of medical resources [18]. Hence, there is a need to explore the adverse impact of DTR on CVD morbidity in under-development rural areas, and investigate the modifying effects of gender and age.

**Fig. 1:**
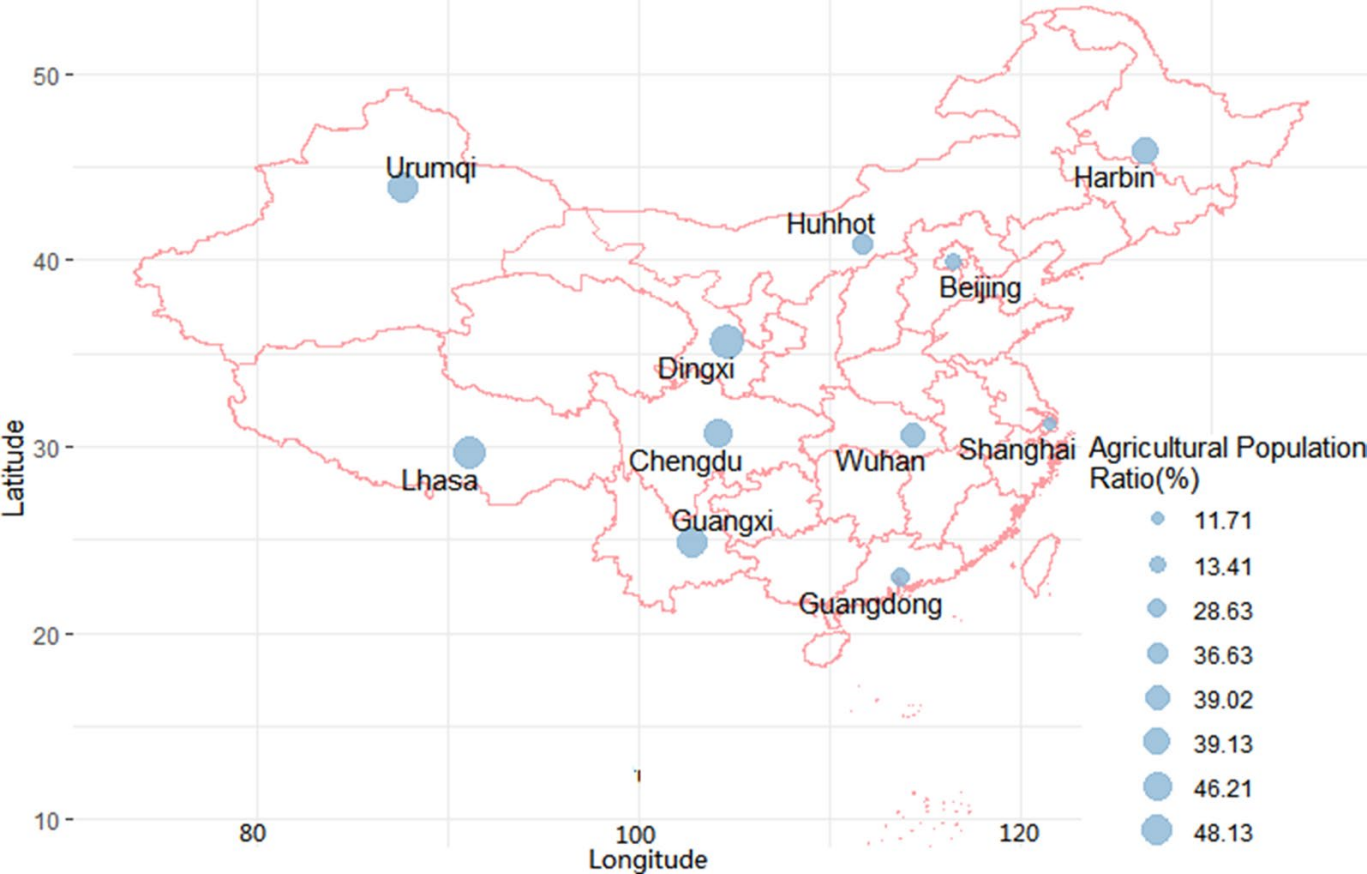
Proportion of agricultural population in Dingxi and major cities in China

This study aimed to investigate the impact of DTR on CVD hospitalization in Dingxi, China, a city dominated by agriculture. Dingxi has the most impoverished counties in China, and 65% of the agricultural population (Fig. 1) [17]. The association between DTR and CVD in Dingxi could help identify susceptible populations and subsequently improve cost-effective preventive strategies. This study stands out from previous studies by evaluating DTR effects on CVD hospital admissions among rural residents in China's most impoverished areas.

## Methods

### Study area

Dingxi (35° 57′ N, 104° 57′ E), a city of Gansu Province, Northwestern China, is one of the poorest regions in China. Meanwhile, Dingxi is located across two climate zones (North temperate sub-humid and semi-arid temperate zone) with poor precipitation, dry climate and severe climate change. It is an important region in the middle of the Silk Road connecting Eurasia. The annual average temperature is 7 °C. The extreme maximum and minimum temperatures are 36.1 °C in summer and − 29.7 °C in winter. In 2019, this district has a population density of 139 persons/km^2^ (Total population = 2,969,900; agricultural population = 2,656,500; land size = 20,330 km^2^; cultivated land size = 5140 km^2^). This district is chosen for three reasons. First, Dingxi has two small climatic zones, which leads to great temperature changes within one day. Second, residents in rural areas experience greater temperature changes, leading to more CVD likelihood [18]. It has the most CVD cases available to our study due to complex climate change. Third, Dingxi is a typical impoverished area in China because it is located in remote areas of China and surrounded by mountains with unfavorable transportation.

### Data collection

The CVD hospital admission data covering the period from January 1, 2015 to December 31, 2019 were collected from the New Rural Cooperative Medical Insurance of Gansu Province (NRCMI). The NRCMI is the government agency in charge of health data collection in Gansu Province. All CVD cases in Gansu Province must be recorded to NRCMI. The 10th edition of the International Classification of Diseases (ICD-10) is used in diagnosis. The CVD patterns included chronic rheumatic heart disease (I05–I09), hypertension (I10–I15), ischemic heart disease (I20–I25) and other types of heart disease (I30–I52). The medical records included information on hospital admissions, age, sex, residential address, and diagnostic ICD codes.

In terms of meteorological data, climate variables were obtained from the China Meteorological Science Data Sharing Service. Data included daily mean temperature, minimum temperature, maximum temperature, relative humidity (%) and atmospheric pressure (hPa). The daily mean temperature was the average of daily maximum and minimum temperatures. Temperature change was measured by the diurnal temperature range (DTR), defined as the intraday difference between maximal and minimal temperature.

### Statistical methods

The distributed lag nonlinear model (DLNM) is suitable to assess the nonlinear and hysteresis effect of exposure on public health. Several epidemiologic studies have successfully used DLNM to evaluate the association between meteorological factors and disease occurrence [19, 20]. To assess the independent impact of DTR on relative risks of CVD, DLNM was applied in this study after considering potential confounders, such as mean temperature, relative humidity, sunshine duration, and average wind speed. The model we used was as follows:

$$\begin{aligned}{\text{Log}}[E({Y_t})]&=\alpha+\beta({\text{DT}}{{\text{R}}_{t,l}})+ns({\text{Humidit}}{{\text{y}}_t},df)+ns({\text{Su}}{{\text{n}}_t},df)+{\text{DOW}}+{\text{Holiday}}\\&\quad+\,ns({\text{Windspee}}{{\text{d}}_t},df)+ns({\text{Temperatur}}{{\text{e}}_t},df)+ns({\text{Time}},df)\\\end{aligned}$$

where t is the day of observation (t = 1, 2, 3…21), E(Yt) represents the daily number of hospitalizations for CVD, α is the intercept, DTR_t,l_ is the "cross-basis" matrix of DTR in DLNM. L denotes the lag days. β indicates the vector of the coefficients for DTR, and ns is a natural cubic spline for controlling potential confounding effects by fitting their df (degree of freedom) trend. The impact of the day of week and holiday was included in the model as DOW and Holiday dummy variables. Time represents the long-term tendency.

Prior studies found that long-term trends, relative humidity, wind speed and sunshine hours were the major contributors to the relative risk of CVD observed in their studies [11, 21, 22]. To estimate the independent impact of DTR on outpatient CVD visits, DLNM with natural cubic splines was applied. Specifically, we used a 3-df natural cubic spline to control the effect of humidity, wind speed and sunshine [23, 24], and 4-df of that to control the impact of mean temperature [25, 26], yet 7-df of that to remove the long-time tendency [10, 23, 25]. Like previous studies [27, 28], the degree of freedom for both DTR and lag in cross-basis matrix ranged from 3 to 6. We used natural cubic spline to define the “cross basis”function with a df 4 and 3 for DTR space and lag space. This was done according to Akaike's information criterion (AIC) by adjusting it for each factor to minimize the AIC value [29, 30] [see Additional file 1]. We set spline knots at equally spaced values within the DTR ranges and intervals in the logarithmic scale of the delays. Also, we defined DOW and Holiday variables as categorical variables to modify their influence. Some air pollutants are also associated with cardiovascular [31]. However, several studies have shown that air pollutants do not necessarily have or have very little effect on main results [32, 33], especially in non-industrial rural areas, the concentration of air pollutant is very low [34]. Moreover, we compared the models with and without air pollutants, and found that there was no difference [see Additional file 2]. In order to avoid overfitting, we didn’t consider air pollutants in our model. The present study gives unbiased effect estimates.

The extremely high and low DTRs were defined as the 5th (4 °C) and 95th (19 °C) percentiles in the DTR distribution. All the results are interpreted as the relative risk (RR) at specific DTR compared to reference DTR (12 °C) with a 95% confidence interval (CI) and 0.05 *p* value. Previous studies suggest that there was a cumulative lag effect of DTR on admission of CVD [24, 35], and most of these studies used lags up to 21 days [25, 36, 37]. Thus, in our study, we considered cumulative lag days of 21 to estimate the delayed effects. We also explored the cumulative hysteresis impact of DTR in different lag days (lags 0–3, 0–7, 0–14, and 0–21). The lag 0-x was used to detect the impact of average DTR in the previous x days on CVD morbidity. Moreover, three-dimensional diagrams were plotted to illustrate the RRs for the relation between DTR and CVD admissions as well as different temperatures and lags by comparing them with the median value of temperature.

To explore the consistency of the results among subgroups, we stratified the study populations by sex (male vs. female), age (using 65 years as the cut-off point). Age 65 years was selected as the cut-off point in age subgroups because it is China's retirement age and people over 65 years are more likely to develop CVD. All the statistical analyses were conducted in 'dlnm' package in the RV3.4.1 statistical software. Finally, we performed sensitivity analyses using different df for DTR, lag in cross-basis and meteorological variables [see Additional file 3–5].

## Results

Weather and CVD hospital admissions data in Dingxi are shown in Table 1. From 2015 to 2019, a total of 24,940 cases occurred, of which 42% were males and 58% were females. Patients aged < 65 years old accounted for 58%. The mean DTR was 11.76 °C (range: 1.6 °C to 26.2 °C), while the average temperature was 8.24 °C (range − 17.9 °C to 24.9 °C).

**Table 1:** Summary statistics for daily variables and cardiovascular diseases (CVD) cases in Dingxi, China, 2015–2019

Variable	Sum	Mean	SD	Frequency distribution				
				Min	P (25)	P (50)	P (75)	Max
Mean temperature (°C)	–	8.24	9.55	− 17.9	0.2	9.7	16.7	24.9
Relative humidity (%)	–	58.99	15.28	16	48	61	71	93
Pressure (hPa)	–	810.4	4.17	797.4	807.3	810.2	813.3	823.1
Wind velocity (m/s)	–	1.79	0.59	0	1.4	1.7	2.2	4.5
Rainfall (mm)	–	1.11	3.73	0	0	0	0.1	54.3
DTR (°C)	–	11.76	4.74	1.6	8	12	15.3	26.2
All	24,940	20	13	1	12	17	25	277
Male	10,608	9	6	1	5	7	10	107
Female	14,332	11	8	1	7	10	14	168
< 65 years	14,568	12	9	1	7	11	16	172
≥ 65 years	10,372	8	6	1	5	8	11	105

**Fig. 2:**
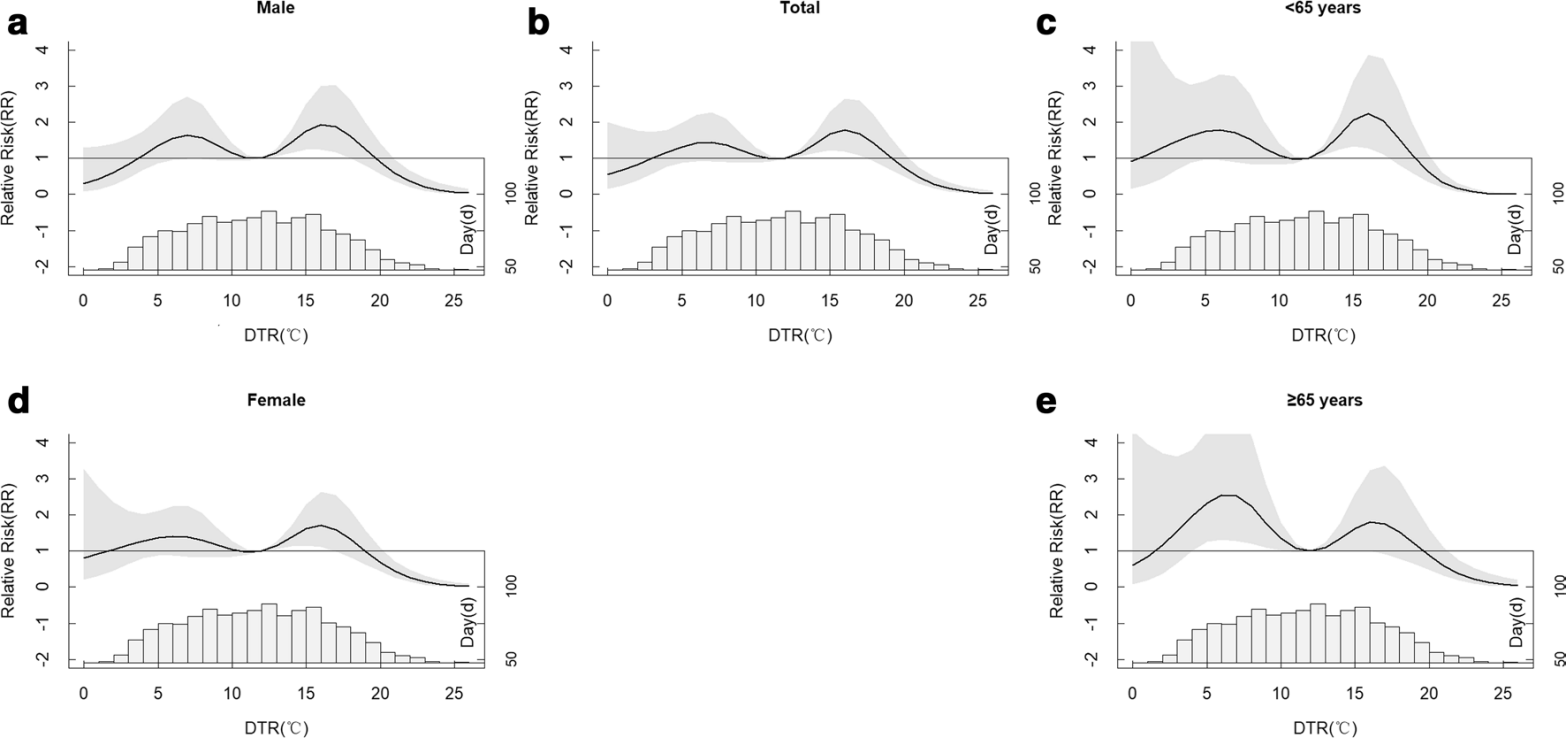
The cumulative lag effects of DTR on CVD’ RR for subgroups. Reference DTR: 12 °C. Lag for 0–21 day

Figure 2 shows the association between CVD and DTR's RR (reference DTR = 12; cumulative days: lag 0–21) in sex and age subgroups. There was a nonlinear relationship (an "M-shape" pattern) between daily DTR and RR of CVD. The first peak effect of DTR on RR was detected at a DTR of 6 °C (RR = 1.418; CI 95%, *p* value < 0.05). The second peak was observed at DTR of 17 °C (RR = 1.781; CI 95%, *p* value < 0.05). These results were consistent across all subgroups. There was a protective effect at extreme DTR (DTR ≤ 3 °C or DTR ≥ 20 °C). Regarding the sex-specific effect modification, the maximum RR at 17 °C (RR 1.937, *p* value < 0.05) in males was slightly higher than females (RR 1.546, *p* value < 0.05). For age-specific effect, the maximum of RR in patients aged < 65 years was observed when DTR was 17 °C (RR 2.233, *p* value < 0.05) and was higher than those aged ≥ 65 years (RR 1.798, CI 95%, *p* value < 0.05). On the other hand, the maximum of RR in patients aged ≥ 65 years was found when DTR was 6 °C (RR 2.549, CI 95%, *p* value < 0.05), and was higher than their counterparts at DTR of 6 °C (RR 1.778, CI 95%, *p* value < 0.05). This indicates that the elderly face more risk of CVD than the adults at the relatively low DTR (6 °C), yet the adults suffer more risk than the elderly at relatively high DTR (17 °C).

**Fig. 3:**
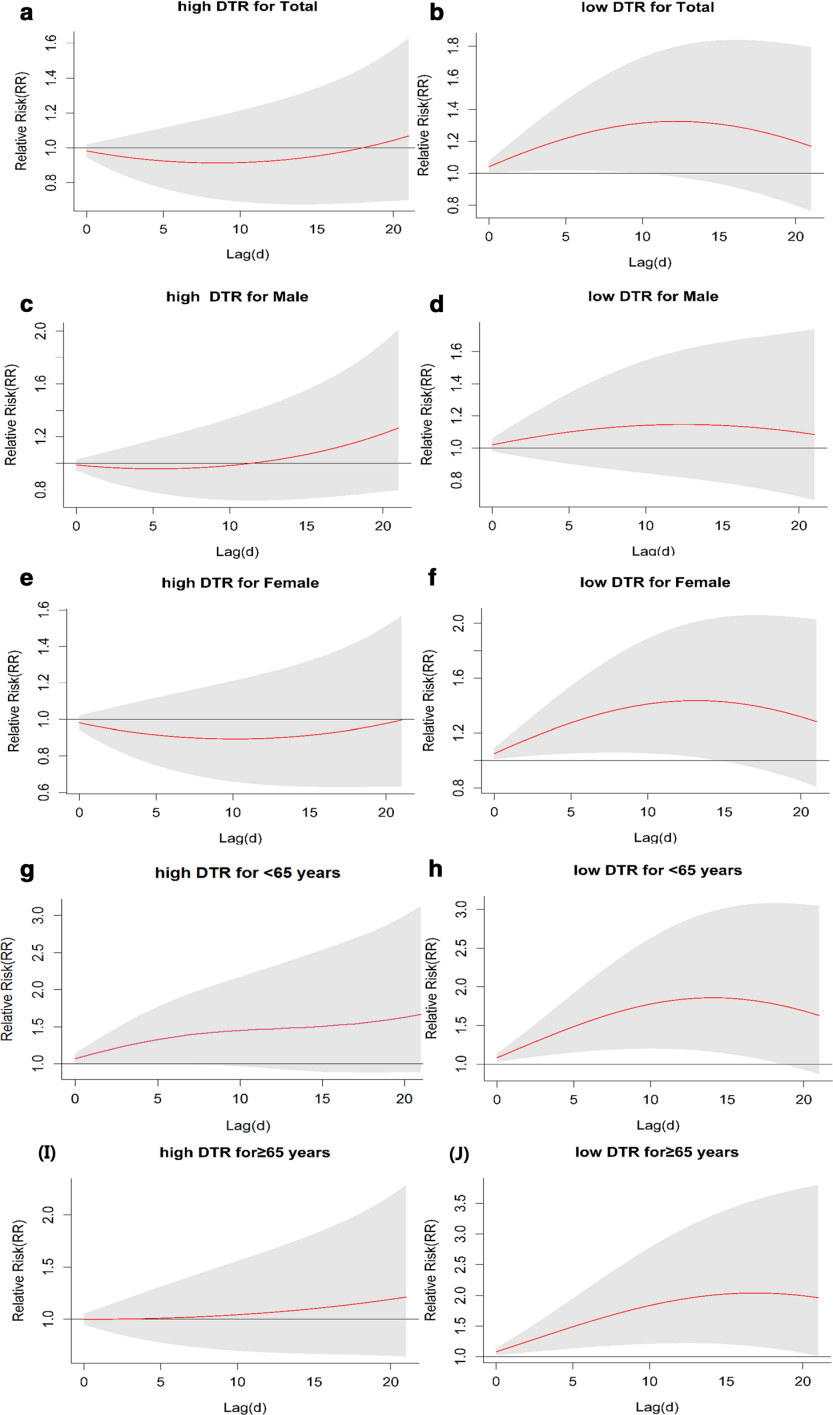
title: Estimated cumulative lag effect of extreme DTR on RR of CVD. Reference DTR: 12 °C. Lag for 0–21 day

Figure 3 presents the estimated 0–21 days cumulative effect of extreme DTR on the RR of CVD with a lag of 0–21 days for the entire, sex-specific, and age-specific study groups (extreme low DTR, 4 °C; extreme high DTR, 19 °C; Reference DTR = 12 °C). In general, the most significant estimated effect of DTR on the hospital admissions of CVD was stronger when DTR was extremely low (RR 1.384 lag day: 14) than high (RR 1.060 lag day: 21). At extremely low DTR (4 °C), the most significant adverse effect of DTR on CVD risk was more pronounced in females (RR 1.438) and elderly (RR 2.034) than males (RR 1.141) and younger adults (RR 1.859) at the 14 days lag. The opposite was observed at extremely high DTR (19 °C); (male: 1.267 females: 0.996; young: 1.586 old: 1.212) at the 21 days lag (Table 2). The effect of extremely low DTR on several CVD cases exhibited a parabola relationship over the day of lag for the entire study subgroups. The greatest effect occurred at a lag of 14 days. As for the impact of extremely high DTR on RR of CVD, there was a protective effect in females. The lag effect occurred after 12 days on males and 18 days in the general population. For age subgroup, the effect of high DTR on CVD cases slightly increased along the lag day.

**Table 2:** Relative risk (RR) of CVD for subgroups at extremely low and high DTR along the lag days

	Extremely low DTR (4 °C, 5th)					Extremely high DTR (19 °C, 95th)				
	Total	Male	Female	Adult	Old	Total	Male	Female	Adult	Old
Lag 0	1.042	1.020	1.051	1.083	1.081	0.982	0.987	0.981	1.087	0.999
	(1.006,1.08)	(0.980,1.061)	(1.011,1.091)	(1.03,1.139)	(1.024,1.14)	(0.946,1.019)	(0.948,1.029)	(0.942,1.021)	(0.985,1.203)	(0.949,1.052)
Lag 1	1.082	1.039	1.1	1.166	1.162	0.966	0.977	0.964	1.126	1.000
	(1.010,1.160)	(0.962,1.121)	(1.022,1.184)	(1.058,1.286)	(1.048,1.289)	(0.899,1.038)	(0.903,1.057)	(0.892,1.041)	(1.000,1.334)	(0.904,1.105)
Lag 3	1.157	1.072	1.192	1.331	1.327	0.941	0.963	0.936	1.257	1.003
	(1.017,1.315)	(0.93,1.236)	(1.04,1.368)	(1.109,1.597)	(1.094,1.61)	(0.823,1.076)	(0.831,1.116)	(0.81,1.081)	(1.002,1.624)	(0.832,1.209)
Lag 7	1.27	1.122	1.342	1.622	1.64	0.915	0.961	0.901	1.395	1.020
	(1.017,1.585)	(0.877,1.435)	(1.059,1.701)	(1.182,2.226)	(1.174,2.293)	(0.727,1.152)	(0.746,1.238)	(0.703,1.156)	(1.010,1.986)	(0.737,1.412)
Lag 14	1.384	1.141	1.438	1.859	2.034	0.941	1.044	0.906	1.445	1.088
	(0.953,1.828)	(0.798,1.643)	(1.012,2.031)	(1.161,2.975)	(1.217,3.292)	(0.675,1.312)	(0.725,1.503)	(0.633,1.296)	(0.916,2.463)	(0.672,1.762)
Lag 21	1.17	1.084	1.283	1.629	1.964	1.060	1.267	0.996	1.586	1.212
	(0.764,1.794)	(0.675,1.741)	(0.811,2.028)	(0.872,3.045)	(1.014,3.801)	(0.7,1.629)	(0.797,2.012)	(0.632,1.569)	(0.931,3.025)	(0.644,2.285)

Reference DTR: 12 °C; *p value < 0.05; CI 95% (Table 2 should appear at line 191 in text)

**Fig. 4:**
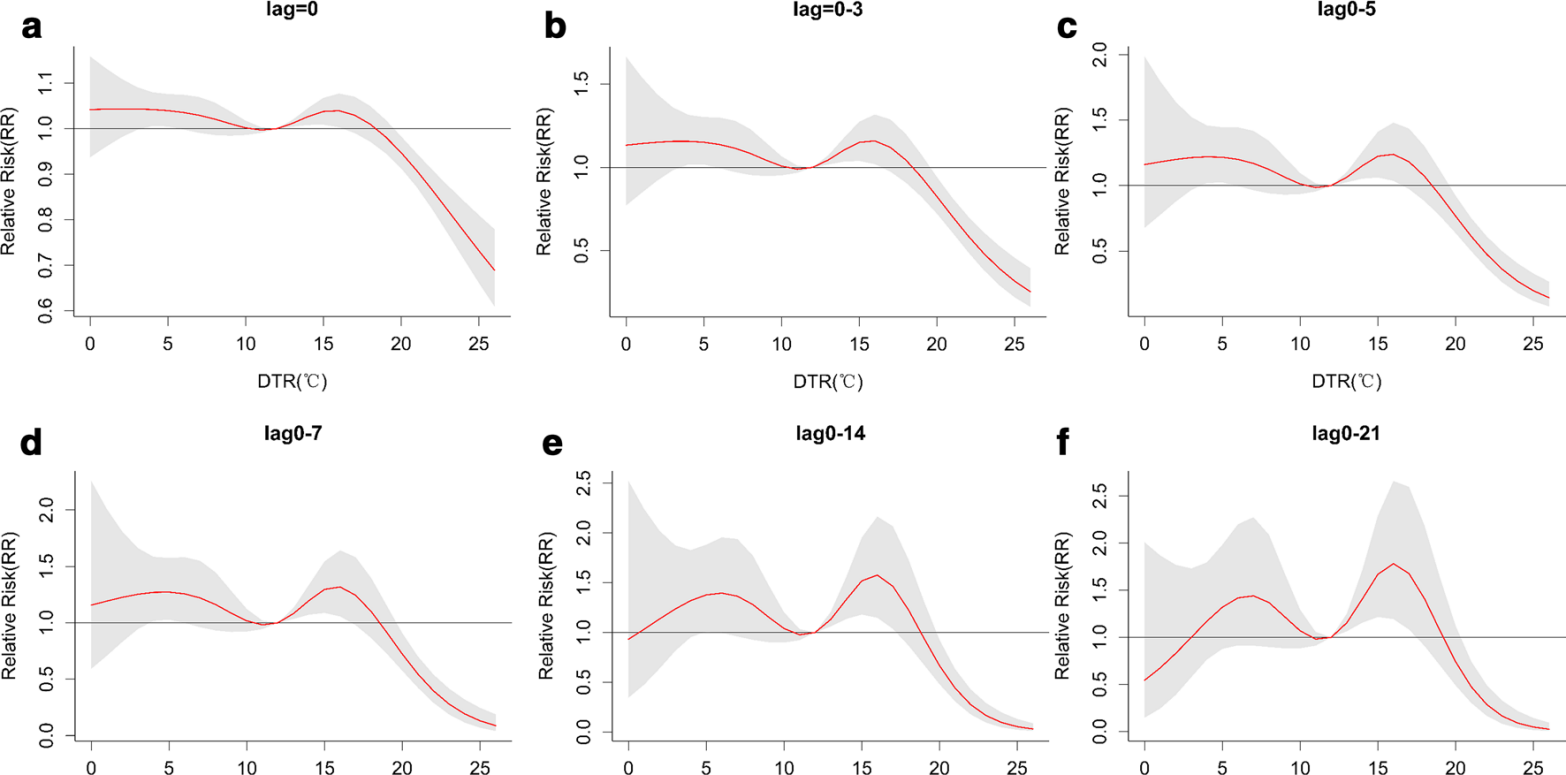
Cumulative effect of DTR on RR of CVD at six different types of lags

Figure 4 illustrates the six different types of the cumulative effect of DTR on the number of outpatient visits for CVD. The morbidity of CVD showed an "M-shape" trend with DTR (with extreme points observed at 6 °C and 17 °C) in six different types of cumulative effects. Cumulative RR of DTR had a gradual enhance along the cumulative day and reached the maximum at a lag of 0–21 days.

**Fig. 5:**
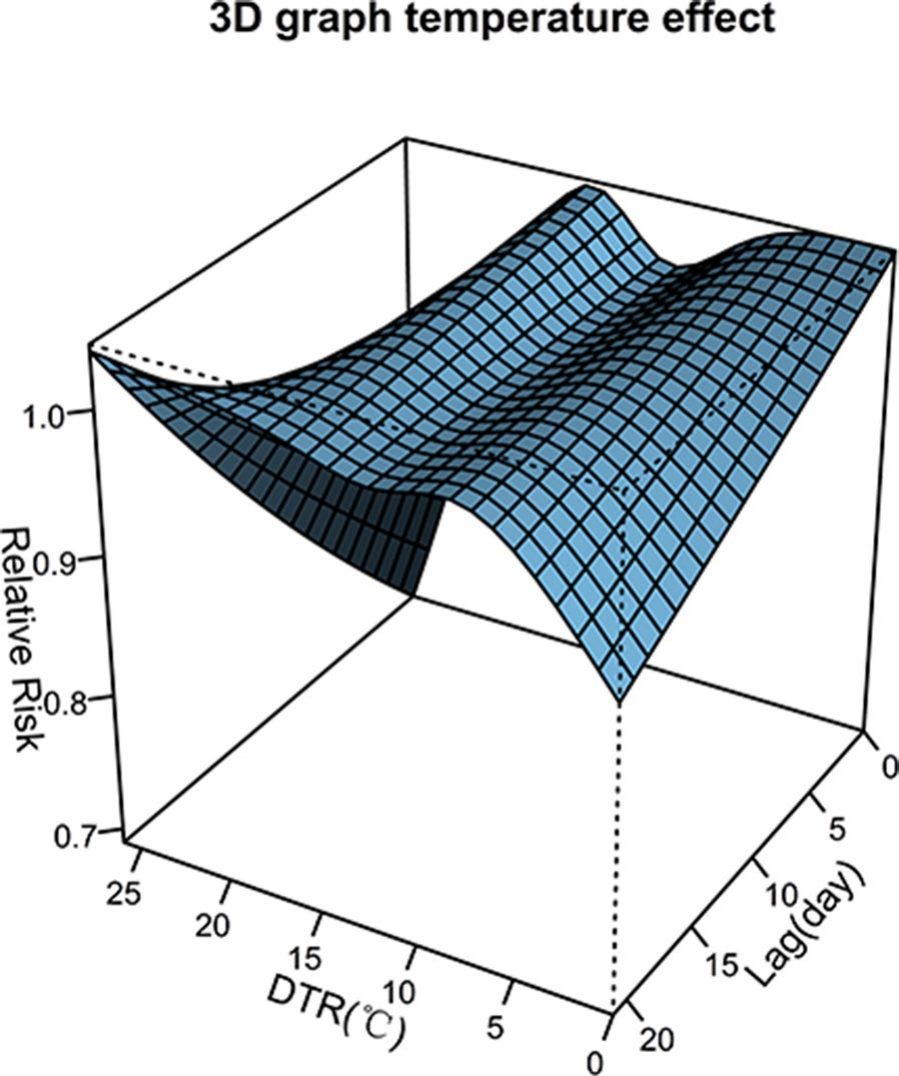
Three-dimensional plot of the relationship between DTR and CVD hospital admission over 21 lag days

Figure 5 depicts a three-dimensional plot to reveal the cumulative effect of DTR on the relative risk of CVD along both the DTR and lags by comparing them with reference median DTR (12 °C). As for the impact of different DTR, the increase of relative risk for CVD was fastest on the lag of 21 days at DTR, ranging from 1.6 to 6 °C. The decrease of that was fastest on the current day at DTR, ranging from 17 °C to 25 °C. The maximum RR for CVD occurred when DTR was 17 °C at lag 21 days (RR 1.78; *p* value < 0.05). The minimum of that occurred when DTR was 26 °C at lag 19 days (RR 0.02; *p* value 0.05).

## Discussion

In this study, we examined the relationship between DTR and the outpatient visits for CVD. There was an M-shaped nonlinear association between CVD’s RR and DTR. The peak of RR of CVD occurred when DTR was 6 °C (total: 1.418 men: 1.546 women: 1.403 young: 1.778 old: 2.549) and 17 °C (total: 1.781 men: 1.937 women: 1.712 young: 2.233 old: 1.798). Our findings suggest that the effect of DTR on CVD is stronger in females and older patients than males and younger patients. Nevertheless, we showed that males and younger peoples suffer more risk of CVD than females and older peoples in extremely high DTR (19 °C). Extremely low DTR (4 °C) exerted a stronger impact on CVD RR than extremely high DTR (19 °C).

The results of our study support a significant association between DTR and the risk of CVD. In line with previous studies, we observed significant acute effects of DTR on all cardiovascular and cerebrovascular diseases ER admissions among males and females living in Beijing [30] and Northern France [38]. In Hong Kong, an increase of 1 °C in DTR at lag days 0–3 was associated with a 1.7% increase in CVD mortality among elderly [14]. In the United States, K. L. et al. concluded that a 10 °C decrease in average daily temperature led to a 13% increase in fatal, incident, and recurrent coronary events in California [34]. A recently published study conducted in Jingchang, Gansu Province, showed a positive linear correlation between DTR with systolic blood pressure and pulse pressure, but a negative linear correlation between DTR with diastolic blood pressure [39]. These findings have led to speculating that blood pressure triggers the onset of cardiovascular diseases. In addition to blood pressure, oxygen uptake, heart rate, and cardiac workload may also increase CVD risk during exposure to daily temperature range [40].

Many prior studies have demonstrated a linear or nonlinear relationship between DTR and CVD. These included studies revealing positive linear associations between DTR and CVD ER admissions in Beijing [30] and Korea [10, 11]. However, the nonlinear (J-, U-, or inverted U-shaped) relationships of DTR-related CVD admission were shown in China, East Asia and Europe [12, 15, 32, 38]. These results were inconsistent with our study as we found a nonlinear relationship ("M-shape" pattern) between daily DTR and RR of CVD admissions. The patterns of association between DTR and CVD were inconsistent even for cities with similar DTR distributions. The difference can be explained by the fact that the effect of DTR varied according to geography, climatic characteristics, housing types, use of cooling and heating equipment, and the scale of DTR for overall all-cause mortality [12, 17].

Studies in China [15, 27], Korea [10, 11], and Japan [12] have pointed out that the impact of DTR on CVD varies by age and gender. These studies concluded that elderly and females are more susceptible to the negative effects of DTR. In our study, when DTR was low (DTR ≤ 12 °C), our results became in agreement with these results. Elderly appear to suffer more risk of CVD, probably owning to increasing age with a progressive reduction in the physiological ability to sense changes in body temperature, and reduction in the body’s compensatory mechanisms (i.e., shiver or sweat) to regulate temperature [41, 42]. As for females, Basu et al. suggest that the differences in the effect of temperature by gender are the result of location and population [8]. That is why the impact of hot temperature on coronary events was greater for men in San Paulo, while greater for women in Mexico City [43]. Furthermore, the research conducted in Europe by Lancet explained that clothing plays a significant role in explaining sex difference and that there is a biological difference between gender in thermoregulation [44].

However, different from prior studies, a novel finding of our study was that the effect on CVD was more pronounced in men and younger people at extremely high DTR (19 °C) comparing with their counterparts. Dingxi is dominated by agricultural development, and our study population is mainly farmers because our data were obtained from the NRCMS (new rural cooperative medical system). In a Southwest China study, farmers had more risk of CVD mortality at high DTR than non-farmers [16]. Ravallion and Chen attributed these results to poor living conditions caused by low annual income, low educational level, and inferior socio-economic status [45]. Also, the agricultural work may lead to an increased high- exposure of DTR [16]. However, in the countryside of Dingxi, more than 70% of young adults go out of the city to work, and most of them primarily in outdoor construction and manufacturing [18]. At the same time, women and the elderly stay at home and work for farming on suitable weather days [46, 47]. This means that women and the elderly are more flexible and selective at work than men in the face of high-DTR weather. This led to more exposure for young men in high DTR days. A greater likelihood of high DTR exposure in the fields might result in a higher risk of CVD [16, 17]. Moreover, more smoking and drinking in young men, as mediating factors, may contribute to the risk of cardiovascular diseases.

Moreover, we found that an extremely low DTR exerts more impact on CVD in all subgroups than an extremely high DTR, which is in line with prior research in Guangzhou [28]. In contrast, a new study showed that extremely low DTR did not increase cardiovascular outpatient visits in the Mediterranean region [48]. The disagreement is possibly because people will suffer more impact of extremely low DTR with the global temperature change and lack of awareness and measures to withstand the low temperature change. The decreasing trend in DTR was mainly caused by the significant increase in the minimum temperature during night-time [49], particularly in Northeast China [50]. Notably, most people, especially the elderly and women, will stay at home and use heat sources [51, 52].

To the best of our knowledge, this is the first study to investigate the association between DTR and CVD risk in the developing area of Dingxi, Northwestern China. Such a place is a backward area for China and a target for achieving universal healthcare. Our observed relation between DTR and CVD morbidity could guide the local authorities to improve CVD preventive strategies in rural areas. The results were based on data from the NRCMI of Gansu Province, which is credible and validated, and NRCMI recorded the CVD events for farmers.

Inevitably, our study has several limitations as well. First, we didn't include all the poverty-stricken areas, but selected a typical rural poor area as a representative. Other studies need to be conducted to evaluate DTR impact in other rural regions in order to generalize our findings. Second, the impact of personal characteristics was not included in our analysis, such as medical history, personal behaviors, and living conditions due to socio-economic status. Third, it is not accurate to assume all the individuals' exposure to DTR to be similar. In addition, in-door use of heating and air-conditioning would lead to measurement bias in the difference between outdoor and indoor temperature. Finally, the retrospective data collection may also bring about bias from diagnostic and coding inaccuracy.

## Conclusions

This study showed that DTR has an adverse effect on CVD morbidity, particularly in women and the elderly. In contrast, men and the elderly suffer more CVD risk than women and younger adults at extremely high DTR. The extremely low DTR exert a stronger effect on CVD compared with extremely high DTR. These findings from Gansu Province could be used by the local Public Health Department to better understand CVD determinants in underdeveloped rural areas and make decisions to improve healthcare in rural communities.

## Supplementary Information

**Additional file 1.** AIC of model for various df of argvar and arglag in cross-basis.

**Additional file 2.** The models with and without air pollutants.

**Additional file 3.** The sensitivity analyses of df for DTR and lag in cross-basis.

**Additional file 4.** The sensitivity analyses of df for temperature and humidity.

**Additional file 5.** The sensitivity analyses of df for wind speed and sunshine duration.

## Data Availability

The datasets used and/or analysis during the current study available from the corresponding author on reasonable request.

## Abbreviations

DTR: Diurnal temperature range
CVD: Cardiovascular diseases
NRCMI: New rural cooperative medical insurance of Gansu Province
DLNM: Distributed lag nonlinear model
RR: Relative risk of CVD
