# Supplementary material for: Impact of diurnal temperature range on cardiovascular disease hospital admissions among Chinese farmers in Dingxi (the Northwest China)
Source: BMC Cardiovasc Disord. 2021 May 22;21:252. doi: 10.1186/s12872-021-02065-8 (PMC8140512; doi:10.1186/s12872-021-02065-8)
Supplement: Supplementary file 1 — Additional file 1. AIC of model for various df of argvar and arglag in cross-basis. [file 12872_2021_2065_MOESM1_ESM.docx]

**Table S1** AIC of model for various df of argvar and arglag in cross-basis

| AIC of model for various df of argvar and arglag in cross-basis | | | | | | | | |
| --- | --- | --- | --- | --- | --- | --- | --- | --- |
| argvar df | 3 | 3 | 3 | 3 | 4 | 4 | 4 | 4 |
| arglag df | 3 | 4 | 5 | 6 | 3 | 4 | 5 | 6 |
| AIC | 10772.4 | 10779.13 | 10805.16 | 10829.66 | 10735.22 | 10750.79 | 10760.51 | 10795.77 |
| argvar df | 5 | 5 | 5 | 5 | 6 | 6 | 6 | 6 |
| arglag df | 3 | 4 | 5 | 6 | 3 | 4 | 5 | 6 |
| AIC | 10757.62 | 10772.7 | 10789.32 | 10829.39 | 10738.13 | 10760.57 | 10789.23 | 10836.57 |

Argvar df represent the freedom of DTR in cross-basis; Arglag df represent the freedom of lag in cross-basis.

**The program of selection df for DTR and lag in cross-basis**

######library######

library(mgcv)

library(dlnm)

library(tsModel)

library(ggplot2)

library(lubridate)

library(Hmisc)

library(reshape2)

library(splines)

library(dplyr)

library(patchwork)

library(bbmle)

library(devtools)

######data######

setwd("C:/Users/lenovo2/Desktop/R ")

#get data

data=read.csv("data.csv",stringsAsFactors =F)

colnames(data)

data$riqi=as.Date(data$riqi)

data$time=as.numeric(data$riqi)

data$DTR=as.numeric(data$DTR)

data$month=month(data$riqi)

length(table(data$year))

range(data$tt,na.rm = T)

y=paste0("V",1)

x=c("tt","AT","rh","localpressure","rainfall","speed","sunshine")

######Select Model method1######

AIC <- data.frame()

for (maxlag in c(14,21,28,30)) {

maxlag <- 21;

df <- 3:6;

list <- as.data.frame(merge(df,df))

for (rowindex in c(1:nrow(list))){

df1 <- list[rowindex,2];df2 <- list[rowindex,1];

argvar <- list(fun="ns",df=df1)

arglag <- list(fun='ns',df=df2)

basis.DTR<-crossbasis(data$DTR,lag=maxlag,argvar=argvar,arglag=arglag)

model <- glm(V1~basis.DTR + ns(daer,7)

+ ns(tt,4)+ ns(rh,4) + as.factor(dow),

family=quasipoisson(),data)

aic<--2*sum(dpois(model$y,model$fitted.value,log=TRUE))+

2*summary(model)$df[3]*summary(model)$dispersion

temp=data.frame(df1=df1,df2=df2,aic=aic)

AIC=rbind(AIC,temp)

}

}

write.csv(t(AIC),'C:/Users/lenovo2/Desktop/R /AIC2.csv')
